# Two key genomic regions harbour QTLs for salinity tolerance in ICCV 2 × JG 11 derived chickpea (*Cicer arietinum* L.) recombinant inbred lines

**DOI:** 10.1186/s12870-015-0491-8

**Published:** 2015-05-22

**Authors:** Raju Pushpavalli, Laxmanan Krishnamurthy, Mahendar Thudi, Pooran M Gaur, Mandali V Rao, Kadambot HM Siddique, Timothy D Colmer, Neil C Turner, Rajeev K Varshney, Vincent Vadez

**Affiliations:** International Crops Research Institute for the Semi-Arid Tropics, Patancheru 502 234, Telangana State, India; Department of Plant Science, Bharathidasan University, 620024 Tiruchirappalli, Tamil Nadu India; The UWA Institute of Agriculture, The University of Western Australia, 35 Stirling Highway, 6009 Crawley, WA Australia; School of Plant Biology, The University of Western Australia, 35 Stirling Highway, 6009 , Crawley, WA Australia; Centre for Plant Genetics and Breeding, M080, The University of Western Australia, 35 Stirling Highway, 6009 Crawley, WA Australia

**Keywords:** Chickpea, Salinity treatment, Quantitative trait loci, Yield, Genomic region, Candidate genes

## Abstract

**Background:**

Although chickpea (*Cicer arietinum* L.), an important food legume crop, is sensitive to salinity, considerable variation for salinity tolerance exists in the germplasm. To improve any existing cultivar, it is important to understand the genetic and physiological mechanisms underlying this tolerance.

**Results:**

In the present study, 188 recombinant inbred lines (RILs) derived from the cross ICCV 2 × JG 11 were used to assess yield and related traits in a soil with 0 mM NaCl (control) and 80 mM NaCl (salinity) over two consecutive years. Salinity significantly (P < 0.05) affected almost all traits across years and yield reduction was in large part related to a reduction in seed number but also a reduction in above ground biomass. A genetic map was constructed using 56 polymorphic markers (28 simple sequence repeats; SSRs and 28 single nucleotide polymorphisms; SNPs). The QTL analysis revealed two key genomic regions on CaLG05 (28.6 cM) and on CaLG07 (19.4 cM), that harboured QTLs for six and five different salinity tolerance associated traits, respectively, and imparting either higher plant vigour (on CaLG05) or higher reproductive success (on CaLG07). Two major QTLs for yield in the salinity treatment (explaining 12 and 17% of the phenotypic variation) were identified within the two key genomic regions. Comparison with already published chickpea genetic maps showed that these regions conferred salinity tolerance across two other populations and the markers can be deployed for enhancing salinity tolerance in chickpea. Based on the gene ontology annotation, forty eight putative candidate genes responsive to salinity stress were found on CaLG05 (31 genes) and CaLG07 (17 genes) in a distance of 11.1 Mb and 8.2 Mb on chickpea reference genome. Most of the genes were known to be involved in achieving osmoregulation under stress conditions.

**Conclusion:**

Identification of putative candidate genes further strengthens the idea of using CaLG05 and CaLG07 genomic regions for marker assisted breeding (MAB). Further fine mapping of these key genomic regions may lead to novel gene identification for salinity stress tolerance in chickpea.

**Electronic supplementary material:**

The online version of this article (doi:10.1186/s12870-015-0491-8) contains supplementary material, which is available to authorized users.

## Background

Chickpea (*Cicer arietinum* L.) ranks second after common bean among the pulses that are consumed [1], and is subjected to various biotic and abiotic stresses during its life cycle. The yield loss in chickpea due to salinity has been estimated to be approximately 8-10% of total global production [2]. Chickpea is known to be sensitive to salinity at both the vegetative and reproductive stages [3], which affects the productivity of the crop across the chickpea growing areas [4]. Despite the sensitivity of the crop to salinity, there is a large variation for salinity tolerance [5-7]. In order to harness the complex phenomenon of salt tolerance, it is important to understand the genetic and physiological basis of salinity tolerance in order to improve existing crop cultivars.

Several studies have been carried out to understand the molecular basis of salt tolerance in other crops and quantitative trait loci (QTLs) for traits associated to salinity tolerance have been identified in cereals like bread wheat [8], barley [9], and in legumes such as *Medicago truncatula* [10], and soybean [11]. In chickpea, the development of molecular markers in recent years has paved the way to dissect the possible underlying tolerance mechanism for various stresses [12]. In chickpea, although several mapping studies have been conducted to identify loci for biotic tolerance [13] and drought tolerance [14] only two studies have reported the presence of QTLs for salinity tolerance [15,16]. Till date very few major QTLs were identified for yield components governing salinity tolerance. Also no major QTL was identified for yield under salinity. Thus it becomes important to identify more number of additional QTLs governing salinity stress tolerance for yield and yield components that can be utilised effectively in marker-assisted genetic improvement of chickpea. Till date there is no report on putative candidate genes that would confer salinity tolerance in chickpea.

The present study reports the analysis of the agronomical traits contributing to increasing yield under salinity, the construction of a genetic map, the use of the agronomical analysis to identify QTLs for yield’ and related traits’ salinity tolerance, and the identification of putative candidate genes using an intra-specific mapping population derived from ICCV 2 (sensitive) and JG 11 (tolerant).

## Results

The detailed results obtained from the unbalanced analysis of variance (ANOVA) for the phenotyping data, such as mean performance of parental lines, range of trait values (i.e., maximum and minimum mean values for each trait) across RILs, broad sense heritability values (*H*^2^), *F* probability values and least significant difference (LSD) of traits across two years and treatments, are provided in Tables 1 and 2.

**Table 1:** **ANOVA results for the parameters evaluated under control and salinity treatments in 2010**

**Control, 2010**									
Trait	Days to flower	Days to maturity	Above ground dry matter (g plant ^-1^)	Yield (g plant ^-1^)	Pod number plant ^-1^	Seed number plant ^-1^	Stem + leaf weight (g plant ^-1^)	Harvest Index	100-seed weight (g)
ICCV 2 (SS)	31	84	22.47	10.86	41.43	41.78	11.61	0.48	25.93
JG 11 (ST)	33	78	24.34	14.18	54.52	60.01	10.16	0.59	23.84
Variation in RILs	23-50	73-99	9.67- 37.35	3.14-18.55	13.97-77.84	27.17-85.21	3.47-19.04	0.18-0.88	14.40-41.58
F Probability	<.001	<.001	<.001	<.001	<.001	<.001	<.001	<.001	<.001
SE	4.63	5.66	5.84	2.89	12.63	13.82	3.35	0.07	2
LSD	9	11	11.49	5.29	24.83	27.17	6.58	0.14	3.94
Heritability (%)	78	61	33	44	43	44	38	71	92
**Salinity, 2010**									
ICCV 2 (SS)	30	69	11.81	5.83	29.08	29.35	5.96	0.49	19.89
JG 11 (ST)	34	81	19.84	10.66	46.79	46.02	8.71	0.57	23.36
Variation in RILs	21-56	63-93	5.23-21.23	2.89-11.02	14.71-62.35	13.69-63.9	2.69-12.16	0.28-1.04	13.64-35.28
F Probability	<.001	<.001	<.001	<.001	<.001	<.001	<.001	<.001	<.001
SE	3.49	4.38	3.14	1.62	6.83	7.04	1.62	0.08	1.74
LSD	7	9	6.17	3.18	13.4	13.81	3.17	0.15	3.42
Heritability (%)	85	80	58	44	59	56	65	58	85

Mean values of nine parameters evaluated (two parents, maximum and minimum mean values from 188 RILs) and *F* probability, standard error (SE), least significant difference (LSD) and the heritability values under control and saline treatment, 2010.

**Table 2:** **ANOVA results for the parameters evaluated under control and salinity treatments in 2011**

**Control, 2011**									
Trait	Days to flower	Days to maturity	Above ground dry matter (g plant ^-1^)	Yield (g plant ^-1^)	Total pod number plant ^-1^	Seed number plant ^-1^	Stem + leaf weight (g plant ^-1^)	Harvest index	100-seed weight (g)
ICCV 2 (SS)	30	76	19.98	10.21	75.97	40.15	9.77	0.53	25.64
JG 11 (ST)	32	79	27.08	14.7	71.34	61.07	12.38	0.54	24.03
Variation in RILs	25-46	73-91	10.55-33.61	4.60-18.13	24.45-109.74	17.59-78.76	5.54-17.42	0.23-0.61	15.17-45.21
F Probability	<.001	<.001	<.001	<.001	<.001	<.001	<.001	<.001	<.001
SE	1.59	2.55	4.18	2.41	14.85	10.13	2.29	0.05	1.65
LSD	3.12	5.01	8.2	4.72	29.14	19.88	4.49	0.11	3.24
Heritability (%)	91	43	52	49	33	49	54	38	91
**Salinity, 2011**									
ICCV 2 (SS)	29	69	9.54	5.92	27.66	23.29	3.62	0.62	25.66
JG 11 (ST)	30	75	13.06	7.14	30.66	29.62	5.92	0.55	24.02
Variation in RILs	23-48	66-88	6.93-25.19	2.91-11.89	11.26-85.12	9.56-54.23	2.45-13.30	0.28-0.71	15.45-44.32
F Probability	<.001	<.001	<.001	0.001	<.001	<.001	<.001	<.001	<.001
SE	2.01	2.17	3.09	1.76	9.57	7.63	1.59	0.05	1.82
LSD	3.95	4.25	6.06	3.45	18.78	14.97	3.13	0.09	3.57
Heritability (%)	90	85	48	40	67	60	64	71	89

Mean values of nine parameters evaluated (two parents, maximum and minimum mean values from 188 RILs) and *F* probability, standard error (SE), least significant difference (LSD) and the heritability values under control and saline treatment, 2011.

### Variance analysis

In both years and treatments the RILs but not the parents showed significant variation for DF (days to first flower) and DM (days to maturity). Parents showed variation for DM in the salinity treatment in both the years. In 2010 with the control treatment, no significant variation was observed between the two parents for all the yield and yield-related traits whereas in the salinity treatment they differed significantly except for the stem + leaf dry weight and the harvest index (HI) (Table 1). In 2011, both the control and salinity treatments did not differentiate the parents for any traits except for filled pod number and empty pod number in the control treatment (Table 2).

The combined unbalanced ANOVA on two years data, for both of the treatments revealed that the traits DF, DM and 100-seed weight were significantly influenced by both genotype and environment, but largely affected by the genetic potential rather than the environment (larger *F* statistic value for the genotype than for the genotype × year component of the variance). All the other traits were influenced significantly by the genotype, but not by the environment component (Additional file 3: Table S3).

### Heritability

Heritability estimates were categorized into low (5-10%), medium (10-30%), high (30-60%) and very high (>60%) according to a previous report [17]. In 2010 in the control treatment, the broad-sense heritability (*H*^2^) of DF, DM, HI and 100-seed weight was high, whereas all other yield and yield-related traits had medium heritability (Table 2). In the salinity treatment, the heritability of DF, DM, 100-seed weight, stem + leaf weight was high, whereas heritability of ADM (above ground dry matter), yield, pod number, seed number and HI had medium heritability values. In 2011, in the control treatment, the traits DF, DM and 100-seed weight had high heritability values, whereas all other traits had medium heritability values (Table 2). In salinity treatment, the traits ADM and yield had medium heritability, whereas all other traits had high to very high heritability values (Table 2). In summary, the phenological traits had high, whereas the yield and yield-related traits had moderate-to-high, heritability values in the salinity treatment.

### Relationships of yield and yield-related traits variables

The seed yield in the salinity treatment correlated significantly to control treatment in both the years (*R*^2^ = 0.23; *R*^2^ = 0.21). Similarly, means of all other traits in the salinity treatment significantly correlated with the control mean of the corresponding trait in both the years (Additional file 4: Table S4). To understand the importance of the QTLs identified, the mean value of traits for which QTLs were found was correlated with the mean yield in both the treatments and across years (Additional file 4: Table S4). Except for DM in the control treatment in 2010 and DF under salinity in 2011, all the other traits for which QTLs were identified showed significant correlations with yield. In the salinity treatment, the ADM, pod number, and seed number explained up to 76%, 75%, and 76% of the variation in yield, respectively. In the control treatment, the stem + leaf weight, filled pod number and seed number explained up to 51%, 56% and 49% variations in yield. Although the HI and the 100-seed weight were significantly correlated to seed yield they explained less than 12% of the yield variation in both treatments [Table 3].

**Table 3:** **Relationship between the traits for which QTLs were identified and yield**

**Control, 2010**		
Days to maturity (DMC1)	CY1 = 0.0616x + 5.2717DMC1	*R* ^2^ = 0.001 (n.s)
Aboveground dry matter (ADMC1)	CY1 = 0.4575x + 0.6915ADMC1	*R* ^2^ = 0.83**
Stem + leaf wt. (ST + LFWTC1)	CY1 = 0.6142x + 3.7464ST + LFWTC1	*R* ^2^ = 0.51**
Harvest index (HIC1)	CY1 = 14.954x + 3.0064HIC1	*R* ^2^ = 0.09**
100- seed weight (100SDWTC1)	CY1 = 0.1337x + 7.1635100SDWTC1	*R* ^2^ = 0.03*
**Salinity, 2010**		
Days to flower (DFS1)	SY1 = 0.0671x + 4.8857DFS1	*R* ^2^ = 0.04**
Days to maturity (DMS1)	SY1 = 0.0915x + 0.2932DMS1	*R* ^2^ = 0.10**
Total pod number (TPDNOS1)	SY1 = 0.193x + 0.7443TPDNOS1	*R* ^2^ = 0.75**
Seed number (SDNOS1)	SY1 = 0.1924x + 0.6744SDNOS1	*R* ^2^ = 0.76**
Harvest Index (HIS1)	SY1 = 11.534x + 1.0604HIS1	*R* ^2^ = 0.12**
100 - seed weight (100SDWTS1)	SY1 = 0.2179x + 2.3611100SDWTS1	*R* ^2^ = 0.11**
**Control, 2011**		
Days to flower (DFC2)	CY2 = 0.4756x + 26.722DFC2	*R* ^2^ = 0.08**
Days to maturity (DMC2)	CY2 = 0.3687x + 75.324DMC2	*R* ^2^ = 0.09**
Aboveground dry matter (ADMC2)	CY2 = 1.6454x + 3.2286ADMC2	*R* ^2^ = 0.85**
Stem + leaf weight (ST + LFWTC2)	CY2 = 0.6454x + 3.2286ST + LFWTC2	*R* ^2^ = 0.48**
Filled pod number (FPDNOC2)	CY2 = 3.034x + 10.336FPDNOC2	*R* ^2^ = 0.56**
Total pod number (TPDNOC2)	CY2 = 2.9113x + 33.653TPDNOC2	*R* ^2^ = 0.28**
Seed number (SDNOC2)	CY2 = 2.9747x + 15.317SDNOC2	*R* ^2^ = 0.49**
100- seed weight (100SDWTC2)	CY2 = 0.7146x + 15.12100SDWTC2	*R* ^2^ = 0.22**
Harvest index (HIC2)	CY2 = 0.0071x + 0.4364HIC2	*R* ^2^ = 0.17**
**Salinity, 2011**		
Days to flower (DFS2)	SY2 = 0.3838x + 27.863DFS2	*R* ^2^ = 0.012(n.s)
Days to maturity (DMS2)	SY2 = 0.6464x + 69.096DMS2	*R* ^2^ = 0.04**
Aboveground dry matter (ADMS2)	SY2 = 1.5322x + 1.2604ADMS2	*R* ^2^ = 0.76**
100 - seed weight (100SDWTS2)	SY2 = 0.5902x + 20.249100SDWTS2	*R* ^2^ = 0.04**
Harvest Index (HIS2)	SY2 = 0.0091x + 0.5234HIS2	*R* ^2^ = 0.04**

All the traits were significantly correlated either at P < 0.001 or P < 0.05 except for days to maturity, control, 2010 and days to flower, salinity, 2011.

As all the traits showed significant correlations between the control and salinity treatments, indicating that the value of traits in the salinity treatment were influenced by the potential value in the control treatment, the traits were expressed as relative values, calculated as the ratio of values in salinity treatment to the mean value of the trait in the control treatment for each RIL. In 2010 and 2011, the relative ADM (*R*^2^ = 0.86, *R*^2^ = 0.76), relative stem + leaf weight (*R*^2^ = 0.52, *R*^2^ = 0.27), relative pod number (*R*^2^ = 0.85, *R*^2^ = 0.64 and relative seed number (*R*^2^ = 0.89, *R*^2^ = 0.89) showed significant correlations with relative yield. This indicated that these traits were important in determining higher yield under salinity in chickpea. By contrast the relative values of phenological traits, 100-seed weight and HI were not significantly related to the relative seed yield (Additional file 5: Table S5).

### Genetic linkage map and marker correspondence

The intra-specific genetic map developed based on ICCV 2 × JG 11 spanned 329.6 cM with 56 markers mapped in 7 out of 8 linkage groups. No markers were mapped on CaLG02. The number of markers mapped per linkage group varied from 2 to 11. On an average one marker/ 5.88 cM were mapped in the present study. The linkage group wise marker correspondence was established between the genetic map constructed in the present study and previously published genetic maps using CMap (Supplementary figure 2 to 10; http://cmap.icrisat.ac.in/cgi-bin/cmap_public/saved_links?selected_link_group=Pushpavalli&action=saved_links_viewer&data_source=CMAP_PUBLIC). There were no common markers between current study and [15,16], but all the three studies had common markers with other published maps that were summarised in Table 4.

**Table 4:** **Linkage group correspondence in three studies to published maps**

**LG number as per published maps**	**Samineni (2010)**	**Vadez et al. (2012)**	**In present study**
LG 1	NA	LG 1 (6)	CaLG01 (3)
LG 2	LG 2 (5)	LG 2 (4)	NA
LG 3	LG 1 (4), LG3 (2)	LG 6 (3)	CaLG03 (3)
LG 4	LG 4 (7)	LG 6 (18)	CaLG04 (3), CaLG05a (3)
**LG 5**	**LG 7 (8)**	**LG 7 (10)**	**CaLG02 (3)**
LG 6	LG 6 (6)	LG 3 (10)	CaLG05b (3)
**LG 7**	**LG 5 (6)**	**LG 5 (7)**	**CaLG07 (6)**
LG 8	LG 8 (4)	LG 4 (5)	CaLG08 (4)

The linkage group number in published maps and the corresponding number in Samineni (2010), Vadez et al. (2012) and in present study were given. The numbers within parenthesis refers to the common markers identified between the linkage group in a population and reference maps. NA- Not applicable. LG 5 and LG 7 in reference maps that harbored salinity tolerance related QTLs across three population were highlighted. (bold + red font).

### QTLs for salinity tolerance

The genotyping and phenotyping data were analysed for identification of major and minor QTLs to understand the genetic basis of salinity tolerance. In the mapping population derived from ICCV 2 × JG 11, a total of 46 QTLs were identified that included 19 QTLs for phenological traits (7 QTLs for DF; 12 QTLs for DM) and 27 QTLs for yield and yield-related traits across years and treatments. The QTL analysis for seven (2010) and nine (2011) yield and yield-related traits detected 23 major QTLs across treatments for all traits (3 QTLs for ADM; 1 QTL for seed number; 1 QTL for pod number; 3 QTLs for yield; 2 QTLs for stem + leaf weight; 9 QTLs for HI; 4 for 100-seed weight) except for filled pod number and empty pod number (Additional file 6: Table S6). In the salinity treatment a few minor QTLs were identified for HI on CaLG04d in 2010, while in the control treatment minor QTLs were identified for yield, pod number, filled pod number and seed number on CaLG07 in 2011.

In case when one of the flanking markers was common to more than one QTL, that region was considered as a single genomic region that contained two or more QTLs. By following this criterion, the 46 QTLs identified were present in 9 genomic regions (Additional file 11: Figure S1). QTLs that contributed >10% of the phenotypic variation explained (PVE) were considered as major QTLs. The PVE by QTLs, in this study, ranged from 6 to 67%. If in a particular treatment, the QTL for a given trait appeared in the same genomic region in more than one year, the QTL was considered as stable QTL [14]. A total of 14 stable QTLs for five different traits in control treatment were identified (Additional file 11: Figure S1).

### QTLs for phenological traits

In 2010, for DF neither in control nor in the salinity treatment major QTL was identified but in 2011, six major QTLs (3 QTLs in the control and 3 QTLs in the salinity treatment), for DF were identified and explained up to 40% of the PVE. In 2010 no major QTL for DM in the salinity treatment was identified but 4 major QTLs (up to 67% PVE) for DF were identified in the control treatment. In 2011, in the salinity treatment, four major QTLs were identified for DM (up to 67% PVE) and in the control treatment; three QTLs (up to 65% PVE) were identified. Four stable QTLs for DM in control treatment were detected, two each in CaLG05 (with flanking markers CaM0463-ICCM272) and in CaLG08 (CKAM1903-CKAM0343) (Additional file 6: Table S6). In any case, since there was no relationship between phenological development and yield either in the control or salinity treatments, these QTLs were not considered important for the primary purpose of this study.

### Yield and biomass

Four yield QTLs (three major and one minor QTL), were identified across two years and treatments. In 2010, in the salinity treatment one major QTL was identified on CaLG07 and explained 17% of the PVE. In 2011, one major QTL in the salinity treatment that explained 12% PVE was also identified on CaLG05, while one major QTL (16% PVE) and one minor QTL (8% PVE) were identified on each of CaLG05 and CaLG07 in the control treatment. The two major QTLs identified in the control and salinity treatments in 2011 were located at the same position on CaLG05 with flanking markers, CaM0463 and ICCM272.

In the salinity treatment, one major QTL for ADM that explained 12% PVE was identified in 2011. In the control treatment, two major QTLs for ADM that explained up to 27% PVE were identified across years. All the three QTLs for ADM were found at the same loci of CaLG05 (CaM0463-ICCM272). Thus two stable QTLs for ADM in control treatment were identified. In the salinity treatment, no QTL for stem + leaf weight was identified, whereas in the control treatment two major and stable QTLs for stem + leaf weight were identified on CaLG05 (CaM0463-ICCM272) across years (Additional file 6: Table S6).

### QTLs for pod number, filled pod number and seed number

In the salinity treatment in 2010, one major QTL for pod number (25% PVE) was found on CaLG07 (CaM2031-CKAM0165) while in the control treatment in 2011, one minor QTL (8% PVE) was found on CaLG07 (ICCM0034-CaM0906). In the control treatment, one more minor QTL for filled pod number (8% PVE) was found on CaLG07. Again on CaLG07, in the salinity treatment in 2010, one major QTL for seed number with 17% PVE and in the control treatment in 2011, one minor QTL (9% PVE) was identified for seed number. These QTLs were of great interest since the correlation analysis above also showed a close relationship between seed and pod number and yield across treatments.

### QTLs for harvest index and 100-seed weight

The QTL analysis identified nine QTLs for HI across years and treatments. In 2010, in the salinity treatment a minor QTL (6% PVE) for HI was identified on CaLG04d while in the control treatment two major QTLs for HI were identified, one each on CaLG05 (46% PVE) and CaLG08 (10% PVE). In 2011, in the salinity and control treatment, three major QTLs per treatment for HI explaining PVE of 30-49% and 32 to 56%, one each on CaLG05, CaLG04d and CaLG08 were identified. Four stable QTLs for HI under control treatment were identified. Four major QTLs for 100-seed weight, one each per treatment and per year, were identified on CaLG05. Three of the four QTLs for 100-seed weight were identified at the same locus of CaLG05 (CaM0463-ICCM272) and explained PVE up to 40%. Two stable QTLs for 100-seed weight under control treatment were identified. The fourth QTL was also identified on CaLG05, but at a different position which explained 17% of the PVE. Again, although these QTLs were significant, they had limited importance for the primary scope of this study since there was only limited or no significant relationship between 100-seed weight or HI and yield in any of the treatments, especially under salinity (Additional file 5: Table S5).

### Genomic regions harbouring QTLs for salinity tolerance identified

**Figure 1:**
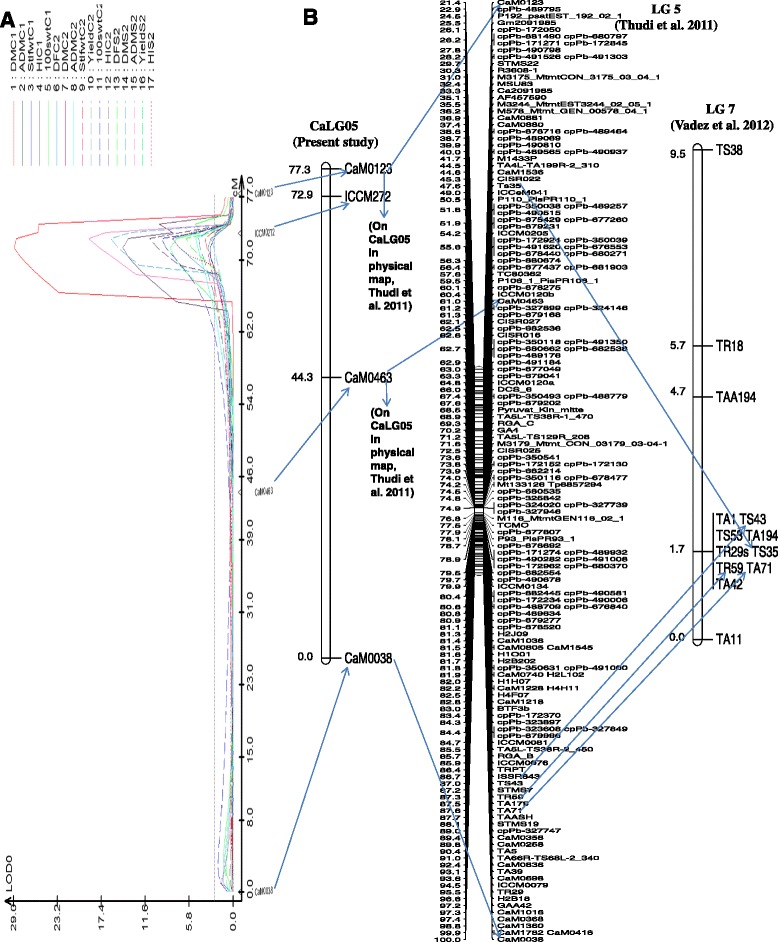
QTLs for seven different traits were identified across years and treatments on CaLG05. **A**. Genomic region on CaLG05 that harboured the 17 QTLs for traits that conferred salinity tolerance in ICCV 2 × JG 11 population were identified using QTL cartographer. **B**. CaLG05 in ICCV 2 × JG 11 population corresponded to LG 5 in Thudi et al. 2011 and LG7 in Vadez et al. 2012.

**Figure 2:**
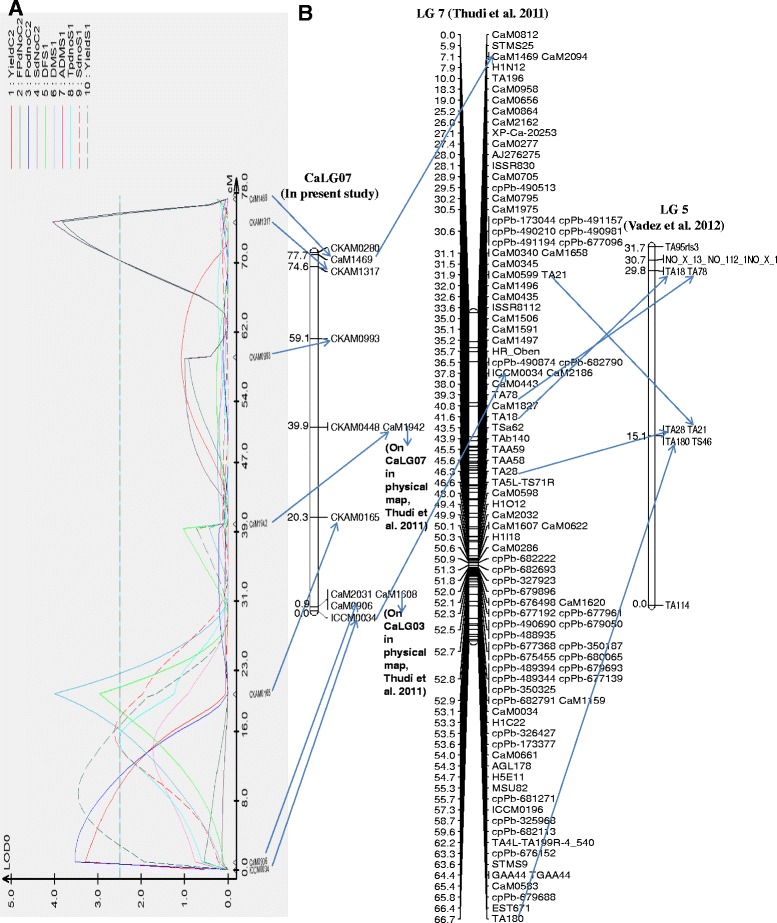
QTLs for five different traits were identified across years and treatments on CaLG07. **A**. Genomic region on CaLG07 that harboured the 9 QTLs for traits that conferred salinity tolerance in ICCV 2 × JG 11 population were identified using QTL cartographer. **B**. CaLG07 in ICCV 2 × JG 11 population corresponded to LG 7 in Thudi et al. 2011 and LG5 in Vadez et al. 2012.

The genomic region of CaLG05 flanked by markers CaM0463 and ICCM272 contained 17 major QTLs for seven different traits (DF, DM, ADM, stem + leaf weight, 100-seed weight, HI and yield) across treatments (Figure 1). Furthermore, one major QTL for DF, DM, ADM, HI, 100-seed weight and yield in the salinity treatment was found in this region. Another genomic region, on CaLG07, harboured seven QTLs, out of which 5 QTLs were identified in the salinity treatment for five different traits (DF, DM, seed number, pod number and yield), but none of these QTLs were stable (Figure 2). A genomic region on CaLG08 harboured eight QTLs (6 in the control treatment and 2 in the salinity treatment) for three traits, DF, DM and HI. Out of these three genomic regions, the genomic regions on CaLG05 and CaLG07 were of greatest interest as they hold QTLs for traits that were significantly related to yield under salinity (Additional file 11: Figure S1).

### Mining candidate genes in salinity stress responsive genomic regions

The BES-SSRs (CaM0463 and CaM0123) on CaLG05 were mapped on Ca5, chickpea reference genome, over a 11.7 Mb (33.1 Mb and 44.8 Mb) distance between the markers. Similarly the BES-SSRs CaM2031 and CaM1942 markers on CaLG07 were mapped on Ca7 over a 12.5 Mb (36.3 Mb and 48.9 Mb) distance between the markers on the chickpea reference genome. A total of 1129 and 440 genes were identified on CaLG05 and CaLG07 respectively (Additional file 7: Table S7). All the identified 1569 genes could be assigned to three functional categories: (i) molecular function, (ii) cellular component and (iii) biological processes.

Though the total number of genes found on CaLG05 and CaLG07 were 1569, the sum of genes assigned to different functional categories (2710) was higher. This is because a given gene may fall in more than one category (Additional file 8: Table S8). In the molecular function category, the highest number of genes fell into binding (575) followed by catalytic activity (501) whereas in cellular component category, the highest number of genes fell into cell part (765) followed by membrane (335). Similarly, in the biological processes category, a maximum number of genes fell into metabolic process (747) followed by cellular process (727) and biological regulation (336) (Additional file 7: Table S7).

Based on gene ontology (GO) annotation, from 1569 genes, 48 putative candidate genes were found to have reported to have a reponse in several plant species to salinity stress (31 on CaLG05 and 17 on CaLG07). These 48 genes were located in a distance of 11.1 Mb (33.6 Mb to 44.7 Mb) and 8.2 Mb (starting at 37.9 Mb and ending at 46.1 Mb) on CaLG05 and CaLG07 respectively.

## Discussion

### Comparing the loci of QTLs for salinity tolerance with previous studies

The genetic map was constructed from ICCV 2 x JG 11 derived population where two key genomic regions related to salinity stress tolerance were identified. To understand whether the genomic regions conferred salinity tolerance across populations, the markers on each LG were compared with published maps and a standard LG number was assigned. For example, nine markers were mapped on LG 5 in a previous report [16]. When we searched for the position of these nine markers in published maps, we found that seven out of nine markers were located on LG 7 in the published maps [18,19]. Thus, the LG 5 was re-assigned to LG 7 to coincide with the published maps. Re-assigning LG numbers was done for each LG group in the three populations (Table 4). By doing this, we were able to compare the key genomic regions identified in the present study with those in the other two studies and this comparison helped us to identify whether a particular LG contained QTLs for salinity tolerance-related traits across populations.

### Genomic region on CaLG05 (CaM0463- ICCM272)

CaLG05 in the present study, LG 7 in [15] and LG 7 in [16] corresponded to LG 5 on the published maps. In the present study on CaLG05, two major QTLs were identified for yield, one in the salinity treatment (12% PVE) and another in the control treatment (16% PVE). The genomic region on CaLG05, flanked by CaM0463 and ICCM272 markers spanning the distance of 28.6 cM, harboured at least one QTL for six different traits per treatment (control, salinity) other than the QTL for yield. So, this locus clearly not only harboured salinity-tolerant QTLs, but also had a highly significant effect on enhancing yield and its related traits across environments in this particular population. Many of the QTLs in that region were found to increase biomass in both treatment and therefore this region would impart increased crop vigour that would eventually lead to a yield benefit. The favourable allele for yield and the QTLs for 6 different traits on CaLG05 were from ICCV 2, the sensitive parent, but known to have good early vigour. In another study, by [15] a minor QTL for yield that explained 8% PVE was located on LG 7 of ICC 1431× ICC 6263 genetic map. In [16], in the salinity treatment the LG 7 of the ICCV 2 × JG 62 mapping population harboured one QTL for seed weight, pod number, HI and 100-seed weight. So after standardising the LG number of three populations, it was clear that the LG 5 of the published maps harboured several important QTLs for salinity tolerance in chickpea (Table 4, Figure 1). Thus, the genomic region found on CaLG05 in the present study (LG 5 in the published maps), is considered to be an important genomic region for future MAB for salinity tolerance in chickpea, and this region appears to confer higher plant vigour.

### Genomic region on CaLG07 in the present study (CaM2031-CKAM0165)

CaLG07 in the present study and LG 5 in [16] corresponded to LG 7 in the published maps. The major QTL that contributed 17% PVE to yield in salinity treatment was identified on CaLG07 using a composite interval mapping approach. In the control treatment a minor QTL (8% PVE) for yield was also found on CaLG07. Two major QTLs for aboveground dry matter on LG 5 (LG 7 as per published maps) with 27% and 20% PVE and also QTLs for HI and DF were identified under salinity conditions by [16]. In the present study, the loci flanked by the markers CaM2031-CKAM0165 on CaLG07 that spanned the distance of 19.4 cM contained one QTL per treatment for yield and pod number.

Unlike on CaLG05, on CaLG07 the QTL for yield that contributed the highest PVE (17%) was found in the salinity treatment, whereas the QTL in the control treatment had a low PVE (7%). The QTL for yield in the salinity treatment in CaLG07 co-maps (at the same position 15.91 cM) with the QTL for pod number and seed number, indicating that this particular loci could be particularly responsible for enhanced yield in salinity stress environments in chickpea, by means of securing a better reproductive success under saline conditions. The allele for the loci is from the salinity-tolerant parent, JG 11 (Figure 2). Therefore, the genomic region found on CaLG07 in the present study is the other important genomic region for future MAB for salinity tolerance in chickpea, and this region appears to confer the capacity to maintain a large number of seeds, probably in relation to an enhanced reproductive success.

### Key traits to impart salinity tolerance

The QTLs for DF and DM were located on CaLG01, CaLG05, CaLG04d, CaLG07 and CaLG08, indicating these traits may be controlled by polygenes present on different chromosomes. Though the phenological traits had high heritability values across treatments and years, there was no significant relationships between the phenological traits and pod yield, so that these QTLs would have no use in breeding salt tolerant lines. Indeed, unlike the study in soybean by [20] phenological traits had no role in determining yield in the ICCV 2 × JG 11 mapping population, this might be due to the fact that both genotypes were early maturing and the range of variation in phenology was small. This was different from an earlier QTL study by [16], in which the two parental lines (one was ICCV 2) had large phenological and yield variation, so that the related QTLs, had to be analysed through the lens of flowering-time differences.

The yield-related traits such as ADM, stem + leaf weight, total pod number and seed number were found to be significantly and linearly related to yield across treatments. Also the mean values of above-mentioned traits in the salinity treatment were significantly explained by the control treatment. So in the mapping population, ICCV 2 × JG 11 used in the present study, QTLs found in the control treatment also holds significant importance in enhancing salinity tolerance. The co-mapping of QTLs for traits like ADM, stem + leaf weight, total pod number, filled pod number and seed number along with the yield QTL makes the two major genomic regions on LG 5 and LG 7 (as per the published maps) promising targets for future breeding of salinity tolerant chickpea.

### Candidate genes identification and its association with salinity tolerance

In plant response pathways to stresses, the membrane receptors, ion channels, histidine kinase etc., perceive the extracellular stress signal and in turn activate complex signalling cascade at intracellular level [21]. This is followed by generation of secondary signal molecules such as Ca^2+^, inositol phosphates; reactive oxygen species (ROS) and abscisic acid (ABA) that transduce stress responsive genes and lead to plant acclimatize for stress tolerance directly or indirectly. The stress induced genes involved in the generation of regulatory molecules like ABA, salicyclic acid and ethylene result in a second round of signalling. These molecules were found to cross talk in stress signalling pathways [21].

The putative candidate genes found in this study were also experimentally demonstrated for their role in salinity stress response by several studies in different plants (Additional file 9: Table S9A and Additional file 10: Table S9B). Across CaLG05 and CaLG07, ten candidate genes that encode for proteins ABA-insensitive 5 like protein, UBP16, HVA22-like, HDA6, and beta glucosidase 24, transcription factors Myb 44, ATHB 5, and GTE10 were identified. These genes were found to have a vital role in ABA biosynthesis, metabolism, and ABA dependent signalling pathways (Additional file 9: Table S9A, Additional file 10: Table S9B). In soybean, novel ion transporter gene *GmCHX1 *was reported to confer salinity tolerance by achieving ion homeostasis [22], something that has been recently hypothesized to potentially play a key role in the adaptation to salt stress in chickpea [65]. In the present study, on CaLG05, three putative candidate genes involved in ion transport encode the proteins of a potassium channel AKT1 (involved in regulating K^+^/Na^+^ ratio), ubiquitin carboxyl-terminal hydrolase 16 and probable inactive poly [ADP-ribose] polymerase SRO2 (regulates plasma membrane antiporter activity) were reported to confer salinity stress tolerance in Arabidopsis [23,24].

Genes involved in the biosynthesis of methionine and osmolytes like Gly betaine were also identified on CaLG05 and CaLG07. Among 48 putative candidate genes, most of the genes were found to play a direct or indirect role in osmoregulation that helps the plants to cope not only with salinity stress but also with other abiotic stresses (Additional file 9: Table S9A and Additional file 10: Table S9B). Identification of putative candidate genes for salinity tolerance on CaLG05 and CaLG07 made theses genomic regions more promising which can be exploited for improving abiotic stress tolerance in chickpea through MAB.

## Conclusions

The present study has identified two potential genomic regions that harboured QTLs linked to salinity tolerance in chickpea and which can be used in MAB. The genomic region on CaLG05 harboured QTLs for six traits in the salinity treatment found to have a role in enhancing productivity across both control and salinity environments, and confers higher plant vigour. Yield and related traits QTLs were also identified in two other populations in the same chromosome region, which validates the importance of that region. The genomic region on CaLG07 harboured major QTLs for yield and its related traits, mainly under salinity, especially seed and pod number. This QTL is hypothesized to confer a higher reproductive success. Availability of chickpea whole genome sequence allowed the identification of putative candidate genes for salinity tolerance in the two genomic regions that were identified, which is being reported for the very first time. The present study opens a window for further research work towards the fine mapping of the genomic regions on CaLG05 and CaLG07 and the identification of novel genes for salinity tolerance in chickpea.

## Materials and methods

### Plant material and treatment conditions

A total of 188 F_8_ RILs were derived from the salt-sensitive parent ICCV 2 and salt-tolerant parent JG 11. The study was conducted in pots buried in the ground at ICRISAT, Patancheru, India (17°30’N; 78°16’E; altitude 549 m). This system enables soil salinity treatments to be imposed in outdoor conditions, but sheltered from the rain [5,6].

Two experiments were carried out between October and February in two consecutive growing seasons (2010-2011 and 2011-2012) with a salinity treatment and a control treatment in both growing seasons. In 2010-2011, the plants were sown on 30^th^ October 2010 and harvested in the first week of February 2011. In 2011-2012, the plants were sown on 25^th^ October 2011 and harvested between 19^th^ January and 6^th^ February 2012 in the salinity pots and between 6 and 25^th^ February 2012 in the control pots. Hereafter, the year of sowing, 2010 and 2011, will be used to indicate the 1^st^ and 2^nd^ experiment, respectively. Maximum temperatures during the growing season ranged from 22 to 32°C in 2010 and 25 to 36°C in 2011, while minimum temperatures ranged from 6 to 22°C in 2010 and 8.6 to 22°C in 2011 with relative humidities of 46-86% during the day in 2010 and 41-79% in 2011.

Pots (0.27 m diameter) containing 7.5 kg of a vertisol (fine montmorillontitic isohyperthermic typic pallustert) soil were buried in the soil so that the outer rim of each pot and outside soil surface were at the same level to avoid direct heating of the pots by solar radiation. The vertisol soil (pH = 8.1, cation exchange capacity (CEC)/clay ratio = 0.87, EC_e_ = 1 dS m^–1^) [17,25] was taken from the ICRISAT farm and fertilised with di-ammonium phosphate at a rate of 300 mg kg^–1^ soil. One-half of the pots were artificially salinized with 1.17 g NaCl kg^–1^ soil, equivalent to 80 mM NaCl in sufficient volume (1.875 L) to wet the vertisol to field capacity. The control pots received tap water containing no significant amounts of NaCl in the same quantity to bring the soil to field capacity. Subsequent watering of both treatments was performed with tap water. The bottoms of the salinized pots were sealed to avoid any salt leaching. Therefore utmost care was taken to water the salt-treated pots, to avoid both water stress and water logging in the pots. To achieve this plants were watered usually every two days, especially at later stage. In our initial work on salt stress, we would estimate the amount of water to be added to reach 90% field capacity with a set of pots weighted at field capacity and then weighted before each watering to assess water losses. Over time and with experience, we would apply a set amount to all pots based on water requirements of the smallest plants, usually every 2-3 days, and then give additional amounts to pots containing larger plants. The watering was also a key element to maintain the salt concentration in the soil solution relatively constant. The pots were also small enough that there was only a very limited salt gradient from top to bottom. In both treatments, six seeds were planted in each pot and later thinned to four similar-sized plants per pot. The experimental design was a randomised block design (RBD) with two treatments, a control (0 mM NaCl) and a salinity treatment (80 mM NaCl) as main factors and genotypes as sub-factors with four replications per treatment (each replicate was a single pot containing four plants).

### Parameters evaluated

The RIL population along with parents was phenotyped for days to 50% flowering (DF) and maturity [DM; in days after sowing (DAS) and recorded when at least two plants per pot commenced flowering or reached maturity]. At maturity, all plants were harvested and oven dried at 65°C for 48 h. After oven-drying, seven yield-related traits - aboveground dry matter g plant^-1^ (including stem, leaves left at maturity and the pods) (ADM), stem + leaf weight g plant^-1^, total pod number plant^-1^, seed number plant^-1^, yield (seed weight) g plant^-1^ were recorded. Harvest Index (HI) was calculated by dividing yield by ADM. The100-seed weight was calculated by dividing yield by seed number and multiplied by 100. In 2011, along with above-mentioned traits, the number of filled pods plant^-1^ and number of empty pods plant^-1^ was counted. Any pod that had no or non-viable seeds was considered as an empty pod. The filled pod number was the difference between the total pod number and the empty pod number. All parameters were measured on a pot basis and calculated on a per plant basis.

### PCR and marker analysis

A total of 98 markers (68 SSRs and 30 SNPs) distributed equally on the chickpea genome were chosen from published genetic maps [18,19,26] for assessing parental polymorphism (ICCV 2 and JG 11). Polymorphic markers were genotyped on the RILs using the polymerase chain reaction (PCR) amplification condition described earlier [18,26]; (Additional file 1: Table S1 and Additional file 2: Table S2). In brief, polymerase chain reactions for all SSR markers were performed in 5 μL reaction volume employing GeneAmp® PCR system 9700 DNA thermal cycler (Applied Biosystems, CA, USA). The SNP markers were genotyped as described earlier by [19].

### Construction of genetic map and QTL analysis

A total of 66 polymorphic markers were used to construct the genetic linkage map using Join Map v 4.0 (www.kyazma.nl/index.php/mc.JoinMap) [27]. In order to find the QTLs responsible for the salinity tolerance, composite interval mapping (CIM) was employed using Windows QTL Cartographer version 2.5 [28]. To gain greater insights into genomic regions controlling the salinity tolerance we compared the results from this study with the previously published genetic maps (http://cmap.icrisat.ac.in/cgi-bin/cmap_public/viewer). Hereafter, the different chickpea genetic maps that were used for comparison were collectively referred as “published maps”.

### Identification of genes for salinity tolerance in present study

In order to identify candidate genes, the bacterial artificial chromosome (BAC)-end derived SSR markers (BES-SSRs) present in the QTL region/or flanking the salinity tolerance QTLs whose physical positions [29] were known were subjected to BLAST against chickpea reference genome assembly [30]. The candidate genes in the regions between the markers mapped on the reference genome were retrieved and functionally categorized using UniProt KB database (http://www.uniprot.org/).

### Statistical analysis

The data were analysed with GENSTAT 14.0 (VSN International Ltd., Hemel Hempstead, UK). An unbalanced ANOVA was performed for all observed parameters individually. Differences between mean values of treatments were evaluated using a LSD test at a 0.05 significance level. Linear regressions were fitted using Microsoft Excel 2007 (Microsoft Corp., 1985, Redmond, Washington, USA). Genotypic and phenotypic components were obtained from ANOVA which was used to calculate the broad sense heritability (*H*^2^).

### Availability of supporting data

All the supporting data are included as a additional files in this manuscript.

## Additiional files

Additional file 1: Table S1. Polymorphic SSR markers used for genotyping the F8 RIL chickpea population of ICCV 2 × JG 11. The unlinked markers are denoted by *. Additional file 2: Table S2. LPolymorphic SNP markers used for genotyping the F8 RIL chickpea population of ICCV 2 × JG 11. The unlinked markers are denoted by *. Additional file 3: Table S3.
*F* probability values (at *P* < 0.01), *F* statistic values obtained with unbalanced ANOVA analysis for genotype, year, genotype*year interaction. Nine and eleven different traits were evaluated under control and saline treatment in 2010 and 2011 respectively. Additional file 4: Table S4. Relationship between the nine and eleven traits evaluated under control and salinity in 2010 and 2011. All the traits were significantly correlated (P < 0.001). Additional file 5: Table S5. Relationship between relative yield in 2010 and 2011 (RY1 and RY2) and relative values of studied parameters. The equations are the fitted linear regressions with the correlation coefficients and level of significance (**-P < 0.01; *-P < 0.05; n.s.- non-significant). Additional file 6: Table S6. Summary of major and minor QTLs for various salinity tolerance related traits. The QTLs were identified using QTL Cartographer on ICCV 2 × JG 11 derived mapping population. Additional file 7: Table S7. 1569 candidate genes with the UniProt Id and protein name found on CaLG05 and CaLG07 were given. Genes that were found to involve in salinity stress response were highlighted (Ca5- Yellow; Ca7-Blue). Additional file 8: Table S8. Gene ontology categorization of 1569 genes identified on CaLG05 and CaLG07. Additional file 9: Table S9A. List of putative candidate genes found to be associated with salinity stress response on CaLG05. (XLS 30 kb) Additional file 10: Table S9B. Table S9B: List of putative candidate genes found to be associated with salinity stress response on CaLG07. Additional file 11: Figure S1. Genetic linkage map of chickpea (ICCV 2 × JG 11) with 56 markers on seven linkage groups. Kosambi map distances are on left- hand side and the genomic regions harboring QTL for salinity-related regions are on right-hand side as listed in Additional file 6: Table S6 in the control and saline treatment, 2010 and 2011.

## Abbreviations

QTL: Quantitative trait loci
SNPs: Single nucleotide polymorphisms
SSRs: Simple sequence repeats
RILs: Recombinant inbred lines
LSD: Least significant difference
DF: Days to first flower
DM: Days to maturity
HI: Harvest index
ADM: Above ground dry matter
ANOVA: Analysis of variance
PVE: Phenotypic variation explained
GO: Gene ontology
LG: Linkage group
MAB: Marker assisted breeding
ROS: Reactive oxygen species
ABA: Abscisic acid
CEC: Cation exchange capacity
RBD: Randomized block design
PCR: Polymerase chain reaction
CIM: Composite interval mapping
BAC: Bacterial artificial chromosome
