# Supplementary material for: Two key genomic regions harbour QTLs for salinity tolerance in ICCV 2 × JG 11 derived chickpea (Cicer arietinum L.) recombinant inbred lines
Source: BMC Plant Biol. 2015 May 22;15:124. doi: 10.1186/s12870-015-0491-8 (PMC4440540; doi:10.1186/s12870-015-0491-8)
Supplement: Additional file 11: Figure S1. — Genetic linkage map of chickpea (ICCV 2 × JG 11) with 56 markers on seven linkage groups. Kosambi map distances are on left- hand side and the genomic regions harboring QTL for salinity-related regions are on right-hand side as listed in Additional file 6: Table S6 in the control and saline treatment, 2010 and 2011. [file 12870_2015_491_MOESM11_ESM.ppt]

## Slide 1
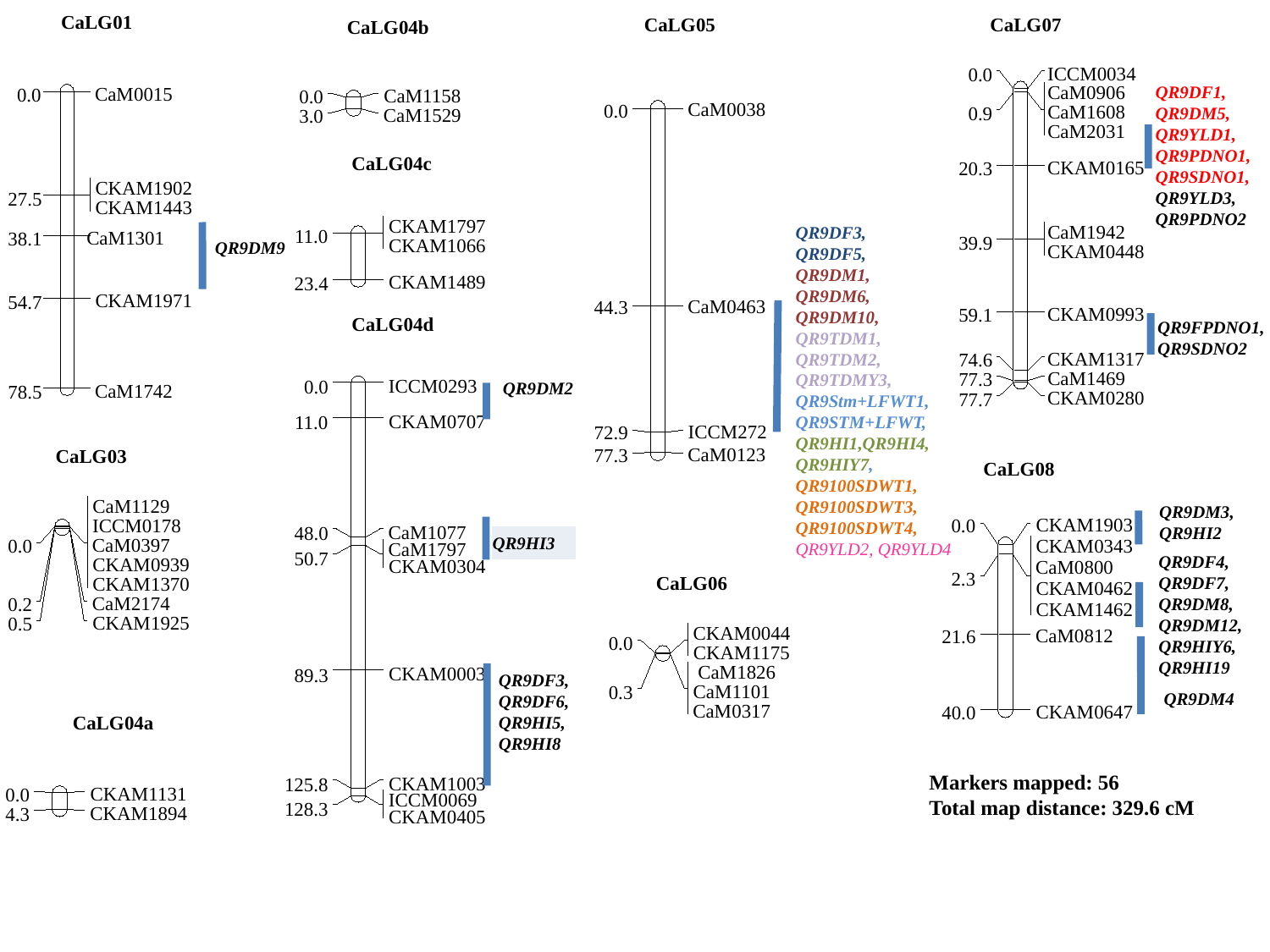

CaLG01
CaM0015
0.0
CKAM1902
27.5
CKAM1443
CaM1301
38.1
CKAM1971
54.7
CaM1742
78.5
CaLG05
CaM0038
0.0
CaM0463
44.3
ICCM272
72.9
CaM0123
77.3
CaLG07
ICCM0034
0.0
CaM0906
CaM1608
0.9
CaM2031
CKAM0165
20.3
CaM1942
39.9
CKAM0448
CKAM0993
59.1
CKAM1317
74.6
CaM1469
77.3
CKAM0280
77.7
CaLG04b
CaM1158
0.0
CaM1529
3.0
QR9DF1,
QR9DM5, QR9YLD1,
QR9PDNO1, QR9SDNO1, QR9YLD3,
QR9PDNO2
CaLG04c
CKAM1797
11.0
CKAM1066
CKAM1489
23.4
QR9DF3,
QR9DF5,
QR9DM1,
QR9DM6,
QR9DM10,
QR9TDM1,
QR9TDM2,
QR9TDMY3,
QR9Stm+LFWT1,
QR9STM+LFWT,
QR9HI1,QR9HI4,
QR9HIY7, QR9100SDWT1,
QR9100SDWT3, QR9100SDWT4, QR9YLD2, QR9YLD4
QR9DM9
QR9FPDNO1,
QR9SDNO2
CaLG04d
ICCM0293
0.0
CKAM0707
11.0
CaM1077
48.0
CaM1797
50.7
CKAM0304
CKAM0003
89.3
CKAM1003
125.8
ICCM0069
128.3
CKAM0405
QR9DM2
CaLG03
CaM1129
ICCM0178
CaM0397
0.0
CKAM0939
CKAM1370
CaM2174
0.2
CKAM1925
0.5
CaLG08
CKAM1903
0.0
CKAM0343
CaM0800
2.3
CKAM0462
CKAM1462
CaM0812
21.6
CKAM0647
40.0
QR9DM3,
QR9HI2
| QR9HI3 |
| --- |
QR9DF4,
QR9DF7,
QR9DM8,
QR9DM12,
QR9HIY6,
QR9HI19
CaLG06
CKAM0044
0.0
CKAM1175
 CaM1826
CaM1101
0.3
CaM0317
QR9DF3,
QR9DF6,
QR9HI5,
QR9HI8
QR9DM4
CaLG04a
CKAM1131
0.0
CKAM1894
4.3
Markers mapped: 56
Total map distance: 329.6 cM
